# Life-history strategy, resource dispersion and phylogenetic associations shape dispersal of a fig wasp community

**DOI:** 10.1186/s40462-017-0117-x

**Published:** 2017-12-06

**Authors:** Vignesh Venkateswaran, Amitabh Shrivastava, Anusha L. K. Kumble, Renee M. Borges

**Affiliations:** Centre for Ecological Sciences, Indian Institute of Science, Bangalore, 560012 India

**Keywords:** Community ecology, Dispersal, Fig wasps, Flight fuel, Insect flight, Life history, Metabolic rate, Resource availability

## Abstract

**Background:**

The combined influence of life-history strategy and resource dispersion on dispersal evolution of a biological community, and by extension, on community assemblage, has received sparse attention. Highly specialized fig wasp communities are ideal for addressing this question since the life-history strategies that affect their pace of life and the dispersion of their oviposition resources vary. We compared dispersal capacities of the wasp community of a widespread tropical fig, *Ficus racemosa*, by measuring flight durations, somatic lipid content and resting metabolic rates.

**Results:**

Wasp species exhibiting greater flight durations had higher energy reserves and resting metabolic rates. “Fast”-paced species showed higher dispersal capacities reflecting requirements for rapid resource location within short adult lifespans. Longer-lived “slow”-paced species exhibited lower dispersal capacities. Most dispersal traits were negatively related with resource dispersion while their variances were positively related with this variable, suggesting that resource dispersion selects for dispersal capacity. Dispersal traits exhibited a phylogenetic signal.

**Conclusions:**

Using a combination of phylogeny, trait functionality and community features, we explain how dispersal traits may have co-evolved with life-history strategies in fig wasps and influenced a predisposition for dispersal. We speculate how processes influencing dispersal trait expression of community members may affect resource occupancy and community assemblage.

**Electronic supplementary material:**

The online version of this article (10.1186/s40462-017-0117-x) contains supplementary material, which is available to authorized users.

## Background

The study of dispersal is important since the ability to disperse can influence the population dynamics of a species, its global distribution, population genetic structure, its evolutionary trajectory, and ultimately its membership within a community [1]. Dispersal capacities have rarely been studied in the context of life-history strategies. One approach to understanding the relationships between life history and dispersal traits in biological communities has been the conceptual framework of competition/colonization trade-offs [2]. However, this framework is helpful only when community members occupy the same guild and compete for the same resource, and fails to capture the complexities of community assemblages within ephemeral microcosms with complicated developmental trajectories.

**Fig. 1:**
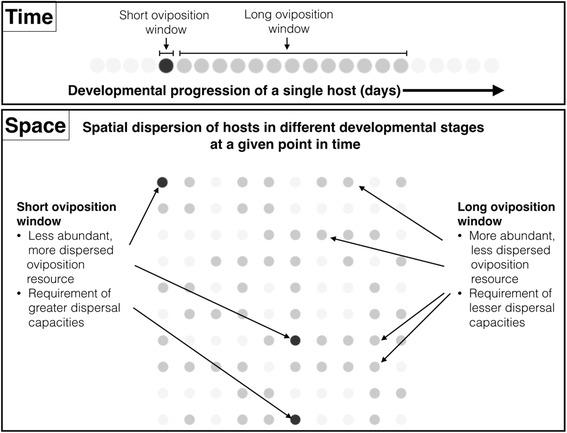
A hypothetical scenario wherein two insect species have oviposition resources on trees that are available for different periods of time (oviposition window). An insect species that can oviposit only during a small time duration (black circles) will have to locate its resources (trees) that are few and dispersed and therefore would require high dispersal capacities. However, an insect species that can oviposit over a larger time duration (dark-grey circles) will locate resources that are more abundant and less dispersed and would require lower dispersal capacities. Thus, the underlying resource dispersion is governed by the length of the oviposition window (length of time during which the tree serves as a resource) despite the same spatial distribution of the host trees

Spatial dispersion of resources can select for dispersal traits of community members [1]. In this paper, we use the terms dispersion exclusively to characterize spatial spread of resources and dispersal to indicate the dispersal capacity of members of a community. When resources appear in a stochastic fashion in space, temporal differences in resource availability can change effective spatial resource dispersion, thereby selecting for corresponding dispersal abilities in community members [3–5]. Specifically, with shorter temporal availability of stochastically occurring resources, the effective spatial resource dispersion increases, selecting for increased dispersal abilities (Fig. 1). Examples of such resource availabilities are found in invertebrate communities that inhabit ephemeral resources such as dung pats, moss patches, phytotelmata, or the enclosed microcosms of fig inflorescences called syconia [6–8]. Although the capacity for dispersal has been recognized as crucially important for membership in such communities [9], no study has attempted to investigate or explain the differences in dispersal capacities of community members. Further, the life-history strategy governing dispersal syndromes in these community members may influence the predisposition to colonize an ephemeral resource/habitat, which may in turn dictate community assemblage and occupancy.

Life-history strategies often fall along a fast–slow continuum [10, 11]. Because a “fast”-paced life history implies time limitation, it may be associated with higher dispersal capacities to reach resource destinations [12, 13]. Broad-scale patterns reveal that traits associated with a “fast”-paced life history (earlier reproductive investment and reduced lifespans) co-occur with high dispersal abilities in bacteria, cancer cells, vertebrates and particularly in aerial ectotherms [14–17]. In insects, a fast-paced strategy is often linked with earlier investment in reproduction as revealed by their ovigeny index (OI): ratio of number of mature to total eggs at eclosion [12, 18]. Often, “fast”-paced life histories are also associated with reduced or complete lack of feeding during adult dispersal stages, with a high reliance on the energy capital acquired during previous life stages [19, 20]. Such co-occurring trait expressions can be viewed as examples of life-history trait syndromes associated with dispersal [21, 22].

Adequately quantifying dispersal traits that dictate dispersal capacity (locomotor capabilities) is essential for understanding dispersal. Estimating realized dispersal abilities in small aerial insects (i.e. < 2 mm in length) is challenging. However, their dispersal capacities can be assessed by measuring dispersal behavior, somatic energy reserves and metabolic rates all of which are dispersal-related life-history traits which, henceforth, we refer to as dispersal traits [23, 24]. Tethered flight performances can also be indicative of flight capacities [25–27]. Capital energy stores (non-polar lipids in the soma) reflect the amount of fuel for flight post eclosion and are especially important for insects incapable of feeding during adult dispersal stages [20, 28]. The mass-specific resting metabolic rate (sRMR) is positively correlated with sustained activity and reflects dispersal flight capacity [29–33]. Variances associated with dispersal traits against the background of ecological selection pressures are informative about the intensity of selection on traits [34]. Therefore, species tracking stochastically appearing resources that are ephemeral and therefore more spread out (higher dispersion) should show higher dispersal trait values and lower associated trait variances compared to species tracking such resources that are less ephemeral and therefore spatially more clumped (lower dispersion). Few studies have measured two or more dispersal traits simultaneously, have studied how they correlate with each other across community members, or have examined the influence of life-history traits in conjunction with resource dispersion within the context of a community (however, see [35] for amphibians).

A valuable model system within which questions about dispersal can be addressed in the context of resource dispersion, life-history strategies and community assemblage is the microcosm of the fig syconium, which is a globular enclosed inflorescence that hosts developing fig wasps. These communities are comprised of wasp species obligately dependent on a single host fig (*Ficus*) species [6, 36]. They consist of a pollinating and a set of non-pollinating wasp species that vary in their life-history traits and oviposition resources within the syconial microcosm [37, 38]. Female wasps oviposit into the syconium, where their offspring develop and mature into adults. Wingless males die after mating within syconia; winged females leave natal syconia and disperse to locate host plants with syconia suitable for oviposition. Tropical fig plants produce syconial crops year-round; they usually exhibit reproductive synchrony (all syconia exhibit the same ontogenetic stage) within plants [39]. Natal plants do not bear oviposition resources when adult females emerge and wasps thus leave their natal plants in search of suitable syconia for oviposition on other fig plants. Different wasp species utilize different niches within the syconium; the earliest arriving wasps use syconium wall tissues and undifferentiated flowers as egg deposition sites; wasps (such as the pollinator) that arrive at the pollen-receptive stage of the syconium oviposit into mature flowers and wasps that arrive after pollen receptivity are parasitoid wasps or inquilines on specific larval/pupal species within the syconium [38, 40]. Each wasp species oviposits into the syconium within a specific time period when the syconium is suitable as an oviposition resource; this is referred to as the oviposition window [37, 41] (Additional file 1: Figure S1). Shorter oviposition windows translate to a smaller proportion of plants bearing suitable syconia of the corresponding ontogenetic stage in a population (Fig. 1). This causes relatively increased oviposition-resource dispersion, which could increase the selection pressure for dispersal and decrease dispersal trait variances compared to longer windows. Therefore, the length of the oviposition window is an important dimension that can affect dispersal abilities. The fig system therefore provides excellent material for an interspecific study of fig-associated wasps— this is due to the differences between wasp species only in the ephemerality of the particular oviposition resources appropriate for each species on the same exploited fig species (Fig. 1).

The context for dispersal in such systems is clear: female fig wasps, after mating within the syconium, disperse to locate specific oviposition resources of different availabilities, offered by a single host species. While wind dispersal is believed to be important for fig wasps, they could be additionally propelling their dispersal through their own dispersal capacities [42–44]. However, there is no knowledge of the active flight capacities of a fig wasp community and their contribution to dispersal. Fig wasps also encounter high mortality during dispersal through predation and temperature/desiccation effects [44, 45]. Therefore, there is likely a high selection pressure for successful dispersal in fig wasp communities. Fig wasp community members also exhibit varying degrees of evolutionary relatedness, thereby allowing the comparative examination of dispersal traits within a phylogenetic context. Fig wasps therefore provide an excellent platform to understand the combined implications of the pace of life and ephemeral resource dispersion on dispersal abilities.

We asked the following questions in a fig wasp community:

1. Do fig wasp species that exhibit a “fast”-paced life-history strategy express traits associated with enhanced dispersal capacities (as shown by longer flight durations, higher somatic lipid content, and greater sRMR) compared to species with a “slow”-paced strategy?
2. Are dispersal capacities negatively related with resource availability in time, i.e. lengths of the oviposition window? Do dispersal trait variances relate positively with this resource availability?
3. How are trait correlations influenced when accounting for phylogenetic relatedness among species? Do dispersal traits exhibit a phylogenetic signal?

## Methods

### Study system

The fruiting phenology of the cluster fig *Ficus racemosa* shows little seasonality and trees exhibit high degrees of within-tree reproductive synchrony but population-level reproductive asynchrony throughout the year [46, 47]. Syconia of *Ficus racemosa* progress through several developmental stages: a) the pre-receptive phase, which constitutes the primordial stages of syconium development and the stages before pollen receptivity; b) the pollen-receptive stage, when pollinators enter syconia to pollinate flowers; c) the post-pollination stage, or the developmental stage when wasps and seeds develop in the syconia; d) the emergence phase, when adult winged female wasps disperse in search of oviposition sites; e) the fruit ripening stage when seeds are dispersed; and f) a gap phase when no syconia are present on the host plant [37] (Additional file 1: Figure S1). The life-history traits, oviposition resource availabilities, trophic position, and niche specialization of all seven wasp species associated with *F. racemosa* are known [37, 48]. Hierarchical clustering methods can be employed to analyze life-history traits as input variables in order to group species based on similarity [37]. Using agglomerative hierarchical clustering and K-means clustering of lifespans and OIs, these wasps can be categorised into two groups that have fast-paced and slow-paced life-history strategies (referred to as “fast” and “slow” respectively); these metrics adequately capture longevities and reproductive effort over their lifespans (Additional file 1: Figure S2). Five wasp species (*Ceratosolen fusciceps*, *Sycophaga stratheni, Sycophaga testacea, Sycophaga fusca* and *Sycophaga agraensis*) cluster in the “fast” group (OI = 1, short lifespans, high fecundity, poor or no feeding abilities) and two wasp species (*Apocrypta westwoodi*, *Apocrypta* species 2) are within the “slow” group (OI = 0.4, long lifespans, low fecundity, feeding abilities) [37]. Their oviposition windows are staggered over syconium ontogeny (Additional file 1: Figure S1). Oviposition window length values were obtained from [37], and vary from (4–19) days in the slow species to (1–5) days in the fast species.

Syconia in late C-phase, i.e. just prior to wasp emergence, were collected from *F. racemosa* plants (*n* = 46) between 2013 and 2015 at the Indian Institute of Science Campus, Bangalore, India (12°58′N, 77°35′E). Wasps were allowed to emerge naturally. If for some reason, wasps did not naturally exit the fig, the syconia was carefully cut open to allow the wasps to exit. Dispersal traits were assessed at the onset of wasp emergence from natal syconia.

### Dispersal traits: Measurement of flight durations

Freshly emerging fig wasps of each species (~2 mm in length) were immobilized on a cool surface and, using a non-toxic synthetic adhesive, were tethered at their thorax at an angle of ~ 45^0^ under a microscope. Upon tethered-flight initiation, wasps were positioned to enable interception of their beating wings by a laser beam of a custom-built optical tachometer (Additional file 1: Figure S3a) whose design was based on [49]. Tethering for flight measurements were performed between 10:00–12:00 h since wasps fly during daytime [50]. Wasps were allowed to perform flight till exhaustion (irreversible immobility). After recording the data in WAV format using commercially available sound cards, the signal was subjected to Fast Fourier Transformation using an audio-processing software to produce spectrograms of the recorded signal. Preliminary analyses of wing-beat frequencies that were conducted before these experiments using stroboscopic and high-speed videography analysis had revealed that the frequency range for wing beats for the different species ranged from 150 Hz–250 Hz. This was used to discriminate the wing-beat signal from spectral noise.

### Dispersal traits: Energy stores in somatic modules

Wasps show considerable variation in size within and across species. Freshly killed frozen wasps of each species were weighed in groups of five. The lipid content in eggs and other reproductive organs reflects reproductive investment [23]. Therefore, all body contents except eggs and organs associated with reproduction such as venom sacs and spermathecae were used for somatic lipid estimation. Five freshly frozen wasps per species were weighed. Afterwards, the somatic and reproductive modules were separated (Additional file 1: Figure S3b) under a drop of phosphate buffer (30 μL) on a glass slide. Then, the somatic components were homogenized with a cold pestle and transferred to a glass vial. Lysis buffer (50 μL) was used to wash the pestle. The non-polar lipid content in the homogenate was estimated using a modification of Foray’s procedure [51]. Organic solvents were handled using Hamilton’s syringes only. To 80 μL of homogenate, 1.4 ml of chloroform:methanol (2:1) solution was added and vortexed for an hour in glass vials. Then, NaCl (200 μL, 0.88%) was added and the mixture was vortexed again for 10 min to allow for the thorough partitioning between aqueous and organic layers [52]. The aqueous layer along with the interstitial fluff was carefully siphoned off, leaving behind the organic layer containing dissolved lipids which was subsequently dried in a vacuum desiccator; 200 μL of chloroform was added and vortexed to allow for lipid solubilization. This solution was used to estimate non-polar lipid content. Tungstosilicic acid (20 mg, dried overnight at 89 °C) was added to 100 μL of this solution and vortexed. Then, the mixture was transferred to a glass insert and centrifuged (9000 rpm, 0 °C, 10 min) to separate the tungstosilicic acid. The chloroform containing the non-polar lipids was then transferred to a vial, dried in a vacuum desiccator, and re-suspended in 100 μL chloroform. Aliquots of this solution were used to estimate non-polar lipid content in somatic modules using the sulpho-phospho-vanillin assay [53]. In a glass vial, 50 μL of the lipid extract was dried in a vacuum desiccator. To this, H_2_SO_4_ (50 μL) was added, vortexed for 10s and placed in a hot water bath at 90 °C for 2 min. Then, the reaction was arrested by placing the material on ice for 5 min and allowing it to attain room temperature for 5 min after which vanillin reagent (200 μL) was added, refluxed using a micro-pipette and was allowed to settle for 5 more min until the development of a stable pink/magenta color. The contents were transferred to a 96-well glass plate, and optical density was recorded by a spectrophotometer at 525 nm. For each reading (representative of five wasps pooled), two technical replicates were obtained, and the mean was used to estimate lipid content of the homogenate. Standard curves were obtained using pure glyceryl trioleate (Sigma-India) stock solutions. The action of tungstosilicic acid was confirmed by performing qualitative (thin layer chromatography) and quantitative (vanillin assay) tests in chloroform mixtures containing known amounts of glyceryl trioleate (non-polar lipid) and phosphatidylcholine (polar lipid) with appropriate controls. Ten measurements were performed per species. For *S. stratheni*, three wasps were used for every homogenate owing to their large sizes (sufficient lipid quantities were present for reliable measurements) and their extreme rarity. Lipid values were divided by the sum of the weights of wasps in the pool to obtain values on a per-wasp basis (μg/unit wet weight).

### Dispersal traits: Metabolic rate measurements

Wasps of each species, in groups of 30, were weighed to obtain wet weights before the estimation of sRMR which was measured using flow-through respirometry [54]. Briefly, a LICOR**™** (Li-820) CO_2_ gas analyzer was calibrated with known percentages of CO_2_. Oxygen gas (~0% CO_2_) at 19 ml/min was used as the flow-through gas and introduced into a 20 ml metabolic chamber (an air-tight polystyrene container) (Additional file 1: Figure S3c). The gas from the metabolic chamber was scrubbed with a dry silica column and passed through two Blue Balston**™** air filters before entering the CO_2_ analyzer which was regularly calibrated against a standard gas mixture (538 ppm CO_2_). For every recording, 30 wasps per species were placed in three perforated PCR vials (10 wasps per vial). Vial perforations were adequate to allow for quick diffusion of gases while small enough to prevent wasp escape. The vials with wasps were then placed inside the larger metabolic chamber which was placed in the dark (wasps reduced movement in the dark, aiding the measurement of sRMR). Afterwards, the readings were taken for 30 min with a 15 min baseline recording before each experiment. Mean CO_2_ output was subtracted from the average baseline value and was considered the CO_2_ output (Additional file 1: Figure S3c). Only for the rare species *S. stratheni*, one to six wasps were used to obtain CO_2_ values based on their availabilities (number of respective samples indicated in parenthesis: 1 wasp (2), two wasps (2), three wasps (1), four wasps (2) and six wasps (1)).

CO_2_ values were normalized per wasp and to average wet weight to obtain sRMR values [55], which are expressed as ml CO_2_/g/h. Experiments were conducted at room temperature (26 °C–28 °C) during the day (8:00 am–12:00 pm). Fifteen readings were obtained per wasp species except for *S. stratheni* for which only eight readings were possible.

Volume of CO_2_ produced was calculated as the product of the flow rate of the gas and the difference between the fractional concentrations of CO_2_ entering and exiting the chamber [53]. The respiratory quotient for all wasps was assumed to be the same, and volume of CO_2_ measured was used directly to make cross-species sRMR comparisons [56].

### Statistical and phylogenetic analysis

To test for statistical differences in the measured dispersal-related life-history traits between the ‘fast’ and ‘slow’ wasp groups, we performed a Mann-Whitney-Wilcoxon test by using pooled trait values of all samples for the “fast” and “slow” species ignoring species identity. We then compared trait values across species using Kruskal-Wallis tests, followed by Dunn’s test to conduct multiple pair-wise comparisons (owing to the underlying non-normality in the residuals revealed using Shapiro-Wilk tests). The significance was tested at an adjusted alpha level of *p* = 0.05 with Bonferroni correction. Next, the trait values were log-transformed and the medians, median absolute deviations, and the coefficient of variation (CV) for each trait were calculated. Linear regression analyses were performed between the log-transformed values of flight duration with somatic lipid content, sRMR, and length of the oviposition window. Linear regressions were also conducted between log-transformed CVs for each dispersal trait and log-transformed oviposition window length. The software R (version 3.2.3) was used for all statistical analyses.

The evolutionary relationships of the *F. racemosa* wasp community were inferred from recent published phylogenetic studies. Chalcidoidea phylogenies have undergone revisions [57–60]; *Ceratosolen* is placed within the Agaonidae, *Sycophaga* within the Sycophaginae, and *Apocrypta* within the Sycoryctinae. In order to infer the evolutionary relationships of the three wasp genera, we used a recent classification of Chalcidoidea in which Sycophaginae are more closely related to Agaonidae than to Sycoryctinae [58]. Additionally, to infer between-species evolutionary relationships of species in genus *Sycophaga* we used information from [47]. The phylogenetic independence was accounted for by calculating phylogenetic generalized least squares (PGLS) [60]. PGLS values were calculated using the *ape* package in R (version 3.2.3). The PGLS values of flight duration were regressed against the PGLS values of lipid content and sRMR. The oviposition window was also considered as a trait (see [61]), and the PGLS values for the oviposition window were calculated and regressed against those of flight duration. Finally, in order to test for a phylogenetic signal, Blomberg’s *K* was chosen because it is based on an underlying Brownian evolution of traits and is well suited for analyses with small sample sizes [62]. The packages *ape*, *phylosignal*, *pgls* and *phytools* in R (version 3.2.3) were used to perform the phylogenetic and associated statistical tests.

## Results

### Life-history strategies and dispersal traits

**Fig. 2:**
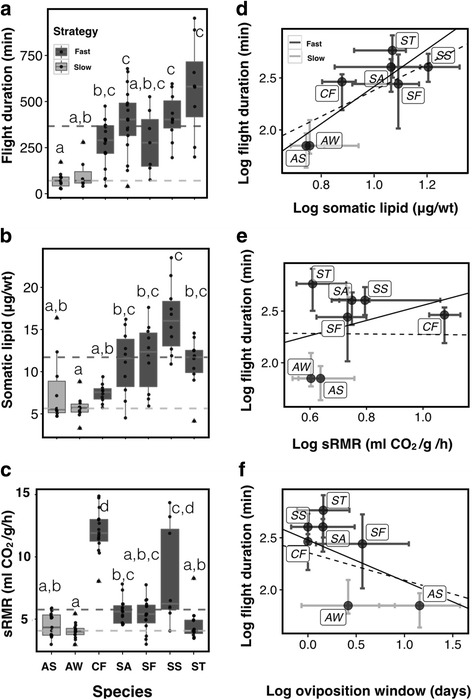
Measured dispersal trait values for fig wasp species along with corrections for phylogeny. **a**, **b** and **c** denote the flight duration, somatic lipid content and sRMR values across species respectively. The black dots indicate data points. The triangles indicate outliers. The horizontal lines in box plots indicate the median. The bottom and top of the box indicate 25th and 75th percentiles respectively while the whiskers indicate either the maximum value or 1.5 times the interquartile range, whichever is smaller. Alphabets indicate significant differences as detected by Dunn’s test for multiple comparisons. The horizontal dotted lines indicate the median value for the groups with fast-paced and slow-paced life-history strategies. **d**, **e** and **f** depict correlations between the log transformed values of time of flight vs. lipid content, sRMR and oviposition window respectively. Plotted are the median values of the transformed parameters with their median absolute deviations represented as line-segments. Solid regression lines indicate associations with the log transformed data, while dashed regression lines indicate the PGLS trends. Species abbreviations: AS-*Apocrypta* species 2, AW-*Apocrypta westwoodi*, SS-*Sycophaga stratheni*, ST-*Sycophaga testacea*, SF-*Sycophaga fusca*, SA-*Sycophaga agraensis*, CF-*Ceratosolen fusciceps*. “Fast” and “slow” indicate the pace of life of the wasp species

Trait values for flight duration, somatic lipid content, and sRMR for each species are provided in Additional file 1: Table S1 (supplementary). The “fast” wasp species had significantly greater flight duration, somatic lipid content (normalized for wet weight), and sRMR than the “slow” wasp species (Flight duration: *U* = 1004, *p* < 0.001; Lipid content: *U* = 846, *p* < 0.001; Metabolic rate: *U* = 1616, *p* < 0.001, Fig. 2a–c).

Pairwise comparisons of dispersal parameter values using Dunn’s test (following significant K-W tests at *p* < 0.05) revealed that flight durations between the two “slow” wasp species (*Apocrypta* sp. 2 and *A. westwoodi*) were not significantly different from each other (Fig. 2a). The three fast wasp species, *Sycophaga stratheni*, *S. testacea* and *S. agraensis*, had significantly higher median flight durations than the wasps in the “slow” category, with *S. testacea* having the longest flight durations. *Sycophaga fusca* and *Ceratosolen fusciceps* had intermediate flight durations, being neither significantly different from the “slow” wasp species or the other three wasp species in the “fast” group (Fig. 2a). The two species in the “slow” group exhibited somatic lipid content values that were similar to each other. Among the species in the “fast” group, *S. stratheni* had the highest lipid content; *S. testacea*, *S. fusca,* and *S. agraensis* had lipid content values that were not different from those of *S. stratheni* or *Apocrypta* sp. 2 but were significantly greater than those of *A. westwoodi*. *Ceratosolen fusciceps* had lipid content significantly lower than that of *S. stratheni* but indistinguishable from those of other wasp species (Fig. 2b).

Species in the “slow” group had sRMR values that were mostly indistinguishable from each other but on the whole had lower sRMR values than species in the “fast” group. The highest sRMR values were for *C. fusciceps* and *S. stratheni. Ceratosolen fusciceps* expressed an sRMR value significantly higher than for all other species except *S. stratheni*. *Sycophaga agraensis* and *S. fusca* expressed similar sRMR values (statistically indistinguishable). *Sycophaga stratheni* exhibited intermediate values but statistically indistinguishable from either *C. fusciceps, S. agraensis* or *S. fusca*. *Sycophaga testacea* expressed sRMR values significantly lower than those for *C. fusciceps* or *S. stratheni. Apocrypta westwoodi* had significantly lower sRMR values than those for *C. fusciceps*, *S. agraensis* and *S. stratheni* (Fig. 2c).

### Dispersal capacity and resource availability

Time engaged in flight and somatic lipid content were significantly positively related (Fig. 2d, *R* ^2^ = 0.7, *p* = 0.01). sRMR and time engaged in flight were weakly but positively associated (Fig. 2e, *R* ^2^ = −0.73, *p* = 0.48). Flight duration and oviposition window were negatively related as expected although this was not significant (Fig. 2f, *R* ^2^ = 0.146, *p* = 0.21). Slope values of the regressions are in Table 1.

**Table 1:** Regression parameters for linear regressions and phylogenetic generalized least square (PGLS) models

	Linear model					Phylogenetic generalized least squares				
	Intercept	Slope	*t* value	*P* value	95% confidence interval	Intercept	Slope	*t* value	*P* value	95% confidence interval
Flight duration ~ lipid content	0.63	1.79	3.88	0.01	0.60–2.97	1.12	1.26	1.5	0.19	−0.39 – 2.9
Flight duration ~ sRMR	1.82	0.73	0.77	0.48	−1.72 – 3.18	2.3	−0.02	−0.03	0.98	−1.64 –1.59
Flight duration ~ Oviposition window	2.48	−0.40	−1.42	0.21	−1.11 – 0.31	2.36	−0.26	−1.59	0.17	−0.59 –0.06

**Fig. 3:**
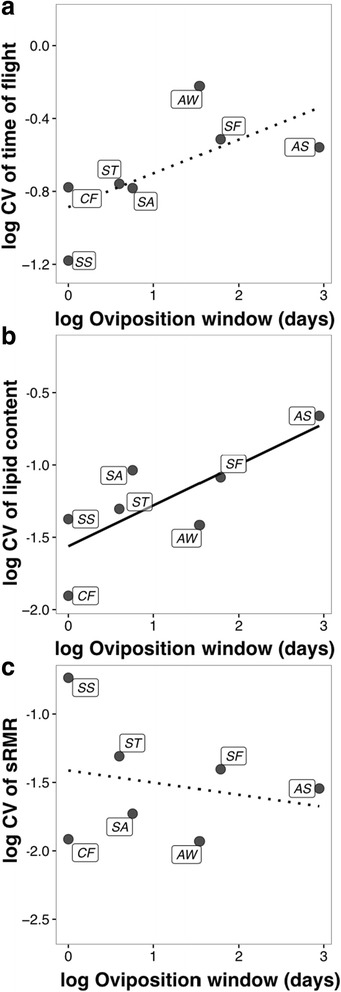
Association of the trait variances (the coefficient of variation) of dispersal trait parameters with oviposition window length for each species. Dashed lines indicate a lack of significance while solid lines indicate significance. Abbreviations of species names are the same as in Fig. 2

Log-transformed CVs for flight durations correlated positively with log-transformed oviposition window length as expected although this was not significant (slope = 0.18; *R* ^2^ = 0.34, *p* = 0.10, Fig. 3a). Log-transformed CVs for somatic lipid content correlated positively and significantly with log-transformed length of the oviposition window (slope = 0.28; *R* ^2^ = 0.53, *p* = 0.04, Fig. 3b). The log-transformed CVs for sRMR, however, were negatively but weakly correlated with oviposition window length (slope = −0.08; *R* ^2^ = −0.13, *p* = 0.62, Fig. 3c).

### Phylogenetic relationships and independence of dispersal trait values

The trend directions of the PGLS regressions were the same as in the linear models for flight durations with lipid content and with oviposition window length; their confidence intervals were also comparable (Additional file 1: Figure S4, Table 1). A phylogenetic signal was observed for flight duration and lipid content, as indicated by Blomberg’s *K* values, which were greater than 1.0 and significant (flight duration: Blomberg’s *K* = 1.56, *p* = 0.04; lipid content: Blomberg’s *K* = 1.93, *p* = 0.0001). Blomberg’s *K* value was also close to 1.0 but not significant for metabolic rates (Blomberg’s *K* = 0.99, *p* = 0.152).

## Discussion

### Life-history and dispersal traits

Fig wasp species differ in their dispersal capacities. “Fast”-paced (time-limited) species displayed significantly higher flight durations, higher somatic lipid content and higher sRMR values than “slow”-paced (longer-lived) species. The duration of the oviposition window (resource availability) was negatively correlated with flight durations. Trait variances of flight durations and lipid content were positively correlated with the length of the oviposition window.

A positive association of flight duration with somatic lipid content (Fig. 2d) suggests a functional role for lipids in aiding flight as observed in other insects [23]. The higher median sRMR values for the “fast” relative to the “slow” wasp species is suggestive of their association with higher dispersal capacities [24, 63]. sRMR values measured are comparable to those published for insects of similar sizes [32]. High sRMR values can lead to the increased accumulation of reactive oxygen species (ROS), thereby decreasing species lifespans [33, 55]; indeed, sRMR values and lifespan are negatively correlated in many insect orders [55, 64]. The highest sRMR values were exhibited by *C. fusciceps* and *S. stratheni*, species that have the shortest lifespans (1 day) [37] and the shortest oviposition windows (1–2 days) (Additional file 1: Figure S1), therefore requiring the highest dispersal capacities. Our results suggest a novel potential negative relationship between dispersal capacity and adult lifespan through sRMR. Life-history strategies, through time limitation, could influence intrinsic dispersal capacities and consequently impact realized dispersal.

Increased dispersal capacities coupled with high OIs (often also coupled with short lifespans and hence time limitation) are optimal when locating highly stochastic resources [12, 19, 65]. The opposite is expected for “slow” species that express OI values less than 1, have longer lifespans, and are presumably less time-limited.

For the “fast” wasp species, the necessity to locate ephemeral oviposition resources, coupled with the inability to feed during dispersal stages (reliance on capital energy reserves), and greater time limitation (owing to shorter lifespans), could require higher dispersal capacities, as indicated by greater pre-dispersal energy reserves, longer flight durations, and higher sRMR values compared to the “slow” ones. Such patterns of life-history and dispersal trait expression are common in many aerial ectotherms [13, 17, 21].

### Dispersal capacity and resource availability

The predicted negative association between flight durations and oviposition window, albeit non-significant, suggests that the latter potentially selects for dispersal traits in fig wasp communities. The non-significance is likely due to the small sample size since there are only seven species in the community. However, the negative slope does suggest that the length of the oviposition window is negatively correlated with dispersal capacities and, therefore, may select for dispersal capacities. Since our study is the first of its kind, we believe that the function of our linear regressions at this stage should be assessed in a descriptive rather than in an inferential manner. More such studies are required to confirm our hypothesis. Such a confirmation could add to our understanding of a relatively unexplored aspect of dispersal evolution in spatially and temporally dynamic resource landscapes. Additionally, the positive correlation between the variances of somatic lipid content and flight durations with the oviposition window suggests relaxed selection when resources are less ephemeral; species with larger oviposition windows exhibited greater dispersal trait variance. Since fig wasps are known to be also passively wind dispersed, it is likely that their dispersal success is governed by both their intrinsic dispersal capacities as well as dispersal conferred by wind. Therefore, dispersal by wind could contribute partly to the observed variance in dispersal traits; however, the impact of wind dispersal on these traits is as yet unknown. Despite this source of unaccounted variance, the trend indicates that dispersal capacity is negatively related to the length of the oviposition window. The correlation between the variance of sRMR with the length of the oviposition window was negative. Metabolic rates are influenced by many functions such as growth, reproduction, age, and maintenance of competent immune systems and may be influenced by myriad selection pressures [66].

### Phylogenetic relationships and phylogenetic signal

PGLS revealed trends similar to the trends obtained from ordinary least square regressions. Specifically, the trends between flight durations, lipid content and oviposition window remained positive and were consistent with expectations. The weakest associations were between metabolic rates and flight durations; the reasons for this may be owing to the same considerations discussed above. A phylogenetic signal for flight duration and lipid content suggests that these dispersal traits are influenced by phylogenetic inertia.

### From dispersal capacities to realized dispersal

Realized dispersal in fig wasps can be governed by an active (dispersal capacity) and a passive element (e.g. wind-assisted movement). Aerial sampling has revealed that fig wasps of monoecious figs (such as the wasp community of *F. racemosa*) are passively wind-dispersed over long distances [42–44]. Population genetic studies show that *C. fusciceps* and *F. racemosa* form a single, largely genetically homogenous population of fig wasps and figs in southeast Asia through long-distance movement of pollen and pollinator genes [67]. Evidence from pollen gene flow suggests that *Ceratosolen arabicus*, the pollinator of *Ficus sycomorus*, can be wind-dispersed over 160 km within its short lifespan of a single day [68]. Investigations on relative dispersal of fig wasps of a community are also few; a population genetic study in *Ficus rubiginosa* showed that the parasitoid *Sycoscapter* of its pollinator *Pleistodontes imperialis* disperses further than the pollinator [69]. An investigation using wing loading values as surrogates for dispersal abilities indicated that dispersal–reproduction trade-offs in fig wasps occupying the same trophic guild enabled community co-existence [70]. We demonstrate, for the first time, that intrinsic dispersal capacities of a fig wasp community are likely influenced by resource availabilities. Our results indicate that intrinsic dispersal capacities (movement owing to active flight and locomotion) are likely vital in conjunction with passive (wind-assisted) dispersal.

### Implications of the dispersal syndrome for community assemblage

In evolutionary time, fig wasp communities assemble with the association of a wasp species with the host plant followed by subsequent niche shifts and/or host shifts [71, 72]. Such associations with the microcosm of the syconium commence with the pollinator followed by the non-pollinators. Fig wasp communities across the tropics also exhibit commonalities in community structure [73–75] and are often unsaturated, especially in the tropics [76]. Unsaturation in fig wasp communities has been attributed to a combination of phylogenetic constraints, co-speciation with hosts and constraints in the ability to colonize ephemeral resources [76]. Therefore, features of dispersal-related life-history traits and effective dispersal may also be common across fig wasp communities. The fast–slow continuum in life-history trait expressions reveals early reproductive investment and short lifespan on one hand, and delayed reproduction and long lifespan on the other. The expression of alternate combinations of such traits (e.g. early reproductive investment and long lifespans) is uncommon [77], possibly because of incompatibilities in trait expression, linked trait expressions or pleiotropy [77–81]. Such processes can lead to the expression of a suite of linked traits and can have implications for the evolution and maintenance of dispersal. The linked expression of traits may reduce the freedom for dispersal trait evolution which can influence community assemblage and subsequent niche shifts or host shifts through dispersal-dependent niche exploitation. This may also explain why fig wasp communities remain unsaturated.

## Conclusions

We show that for the wasp community, flight durations and lipid content are potentially more reliable predictors of dispersal than metabolic rates. We demonstrate that the availability of oviposition resources selects for dispersal traits and influences the associated trait variances. We suggest that dispersal-related life-history traits can be selected for by differences in the dispersion of resources for each species and infer that these small wasp species are capable of propelling their dispersal despite passive dispersal agents like the wind. Through trait co-expression patterns both in fig wasps and in other organisms, we posit the operation of trait syndromes that may constrain dispersal-related life-history traits. Finally, we indicate how phylogenetic conservatism of dispersal traits may exist in the wasp community. This coupled with constrained niche shifts or host shifts point towards two possible scenarios: a) species sorting followed by ecological niche fitting, or b) adaptation in response to selection, that shapes the dispersal traits of each species in the community [82]. The predisposition of certain wasps to exhibit a particular dispersal syndrome (due to constrained trait co-expression) may have restricted them to occupy niches of a suitable resource dispersion characteristic. Therefore, the expression of a particular type of dispersal characteristic or capacity may be key in fig wasp occupancy of available niches within an ephemeral microcosm.

## Additional file

Additional file 1: Table S1. Dispersal-trait parameter values (untransformed means and standard deviations) for each fig wasp species in the *Ficus racemosa* community. **Figure S1**. Length of oviposition windows associated with each fig wasp species in relation to the developmental ontogeny of the syconium. **Figure S2**. Results of the phenogram generated using cluster analysis. **Figure S3**. Methods for quantifying dispersal capacity of fig wasps.** Figure S4**. Putative phylogenetic relationships of wasp species of *F. racemosa*. (DOC 778 kb)
