# Supplementary material for: Life-history strategy, resource dispersion and phylogenetic associations shape dispersal of a fig wasp community
Source: Mov Ecol. 2017 Dec 6;5:25. doi: 10.1186/s40462-017-0117-x (PMC5718022; doi:10.1186/s40462-017-0117-x)
Supplement: Additional file 1: Table S1. — Dispersal-trait parameter values (untransformed means and standard deviations) for each fig wasp species in the Ficus racemosa community. Figure S1. Length of oviposition windows associated with each fig wasp species in relation to the developmental ontogeny of the syconium. Figure S2. Results of the phenogram generated using cluster analysis. Figure S3. Methods for quantifying dispersal capacity of fig wasps. Figure S4. Putative phylogenetic relationships of wasp species of F. racemosa. (DOC 778 kb) [file 40462_2017_117_MOESM1_ESM.doc]

**Table S1**: Dispersal-trait parameter values (untransformed means and standard deviations) for each fig wasp species in the *Ficus racemosa* community. OW (oviposition window) = mean and standard deviation (in parentheses) of the length of the oviposition window.

| **Species** | **Flight duration**  **(minutes)** | **CV^†^** | **Somatic lipid (μg/wt)** | **CV** | **sRMR (ml CO_2_/g/h)** | **CV** | **OW** |
| --- | --- | --- | --- | --- | --- | --- | --- |
| *Apocrypta* species 2 | 74.08±42.4 (11) | 0.57 | 7.63±3.94 (10) | 0.52 | 4.54±0.97 (15) | 0.21 | 19.03 (10.3) |
| *Apocrypta westwoodi* | 106.35±85.16 (7) | 0.80 | 5.75±1.4 (10) | 0.24 | 4.04±0.59 (15) | 0.15 | 4.6 (4.9) |
| *Ceratosolen fusciceps** | 270.54±124.34 (18) | 0.46 | 7.44±1.11 (10) | 0.15 | 12.07±1.78 (15) | 0.15 | 1.0 (0.0) |
| *Sycophaga agraensis** | 395.35±180.88 (17) | 0.46 | 11.03±3.91 (10) | 0.35 | 5.62±1.0 (15) | 0.18 | 2.1 (2.7) |
| *Sycophaga fusca** | 283.03±169.28 (7) | 0.60 | 11.74±3.96 (10) | 0.34 | 5.31±1.3 (15) | 0.25 | 6.0 (5.0) |
| *Sycophaga stratheni** | 414.85±127.55 (10) | 0.31 | 16.27±4.12 (10) | 0.25 | 8.42±4.04 (8) | 0.48 | 1.0  (0) |
| *Sycophaga testacea** | 572.89±268.39 (8) | 0.47 | 11.04±3 (10) | 0.27 | 4.49±1.21 (15) | 0.27 | 1.8 (1.6) |

* = fast wasps

**Figure S1.** Length of oviposition windows associated with each fig wasp species in relation to the developmental ontogeny of the syconium. The circle represents the ontogenetic stages of the syconium development. The arcs represent the first and last observed wasp arrivals for oviposition for each species, i.e. the range with respect to the onset of syconium development, and the black regions within the arcs indicate the time when the top 50% of wasps for every species arrived at the syconium. The classification of wasps as "fast" or "slow" based on their life-history strategy is indicated. Wasps begin their development at distinct stages of syconium ontogeny but exit the syconium concurrently. Abbreviations of species names are the same as in Figure 2 (main text).

**
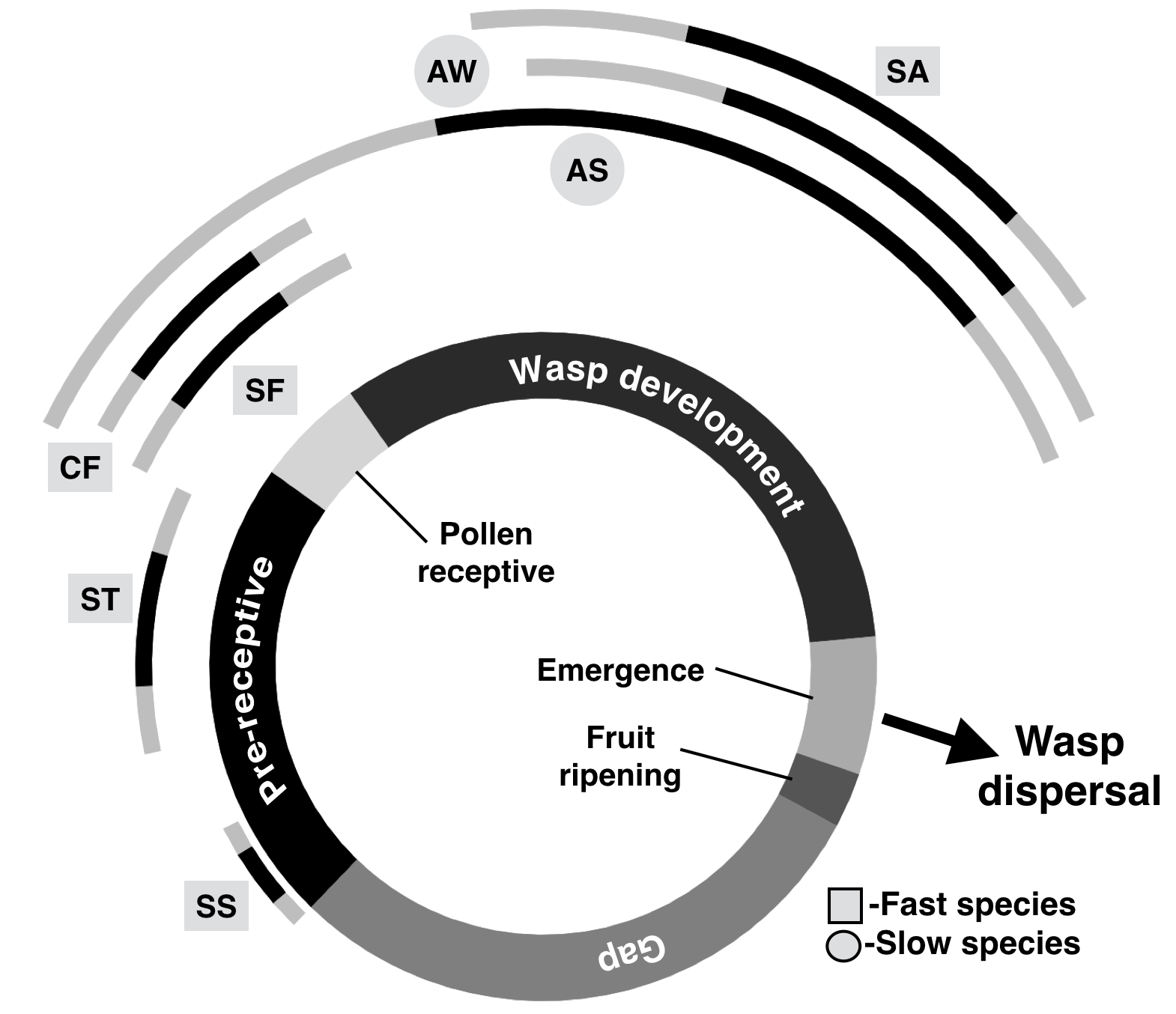
**

**Figure S2.** Figure depicts the results of the phenogram generated using cluster analysis. To identify clusters of wasp species by lifespan and OI, we performed an agglomerative hierarchical clustering of wasp species to generate the trait phenogram using the centroid method. We obtained two distinct wasp groups by the method as depicted. We then employed unsupervised K-means clustering analysis, assigning two clusters in order to check the classification of each species into one of two groups. The procedure was repeated 20 times to ensure reproducibility. The results indicated the same clustering pattern as the one shown in the phenogram. All analysis was performed using the cluster package in the software R (version 3.2.3). We used these clusters as the basis for classifying our wasps into “fast” and “slow” groups. The classification of wasps based on their life-history strategy is indicated. Abbreviations of species names are the same as in Figure 2 (main text).


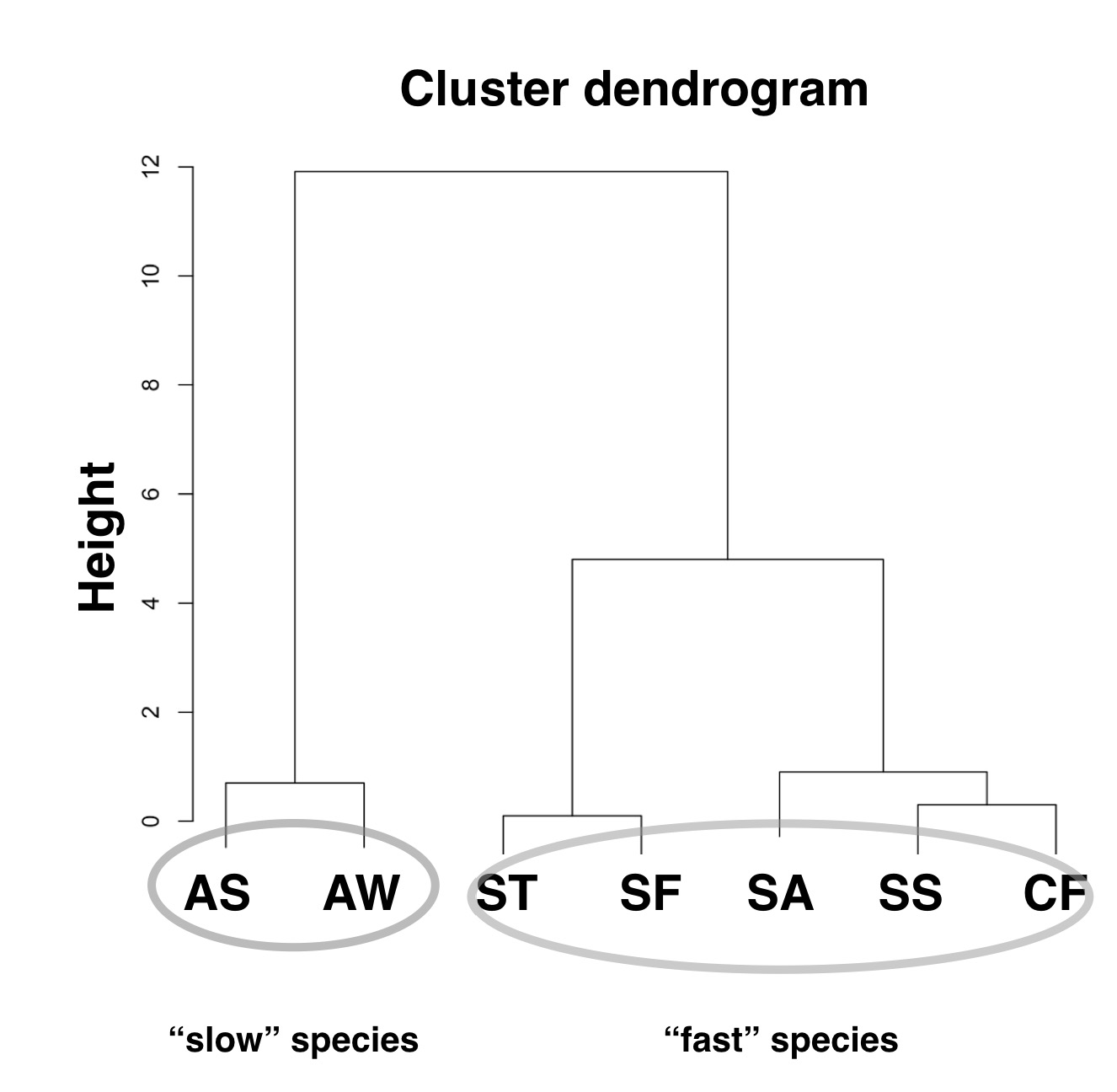


**Figure S3.** Methods for quantifying dispersal capacities of fig wasps. (*a*) tethered flight duration analysis, (*b*) estimation of somatic non-polar lipid, (*c*) estimation of resting metabolic rates.


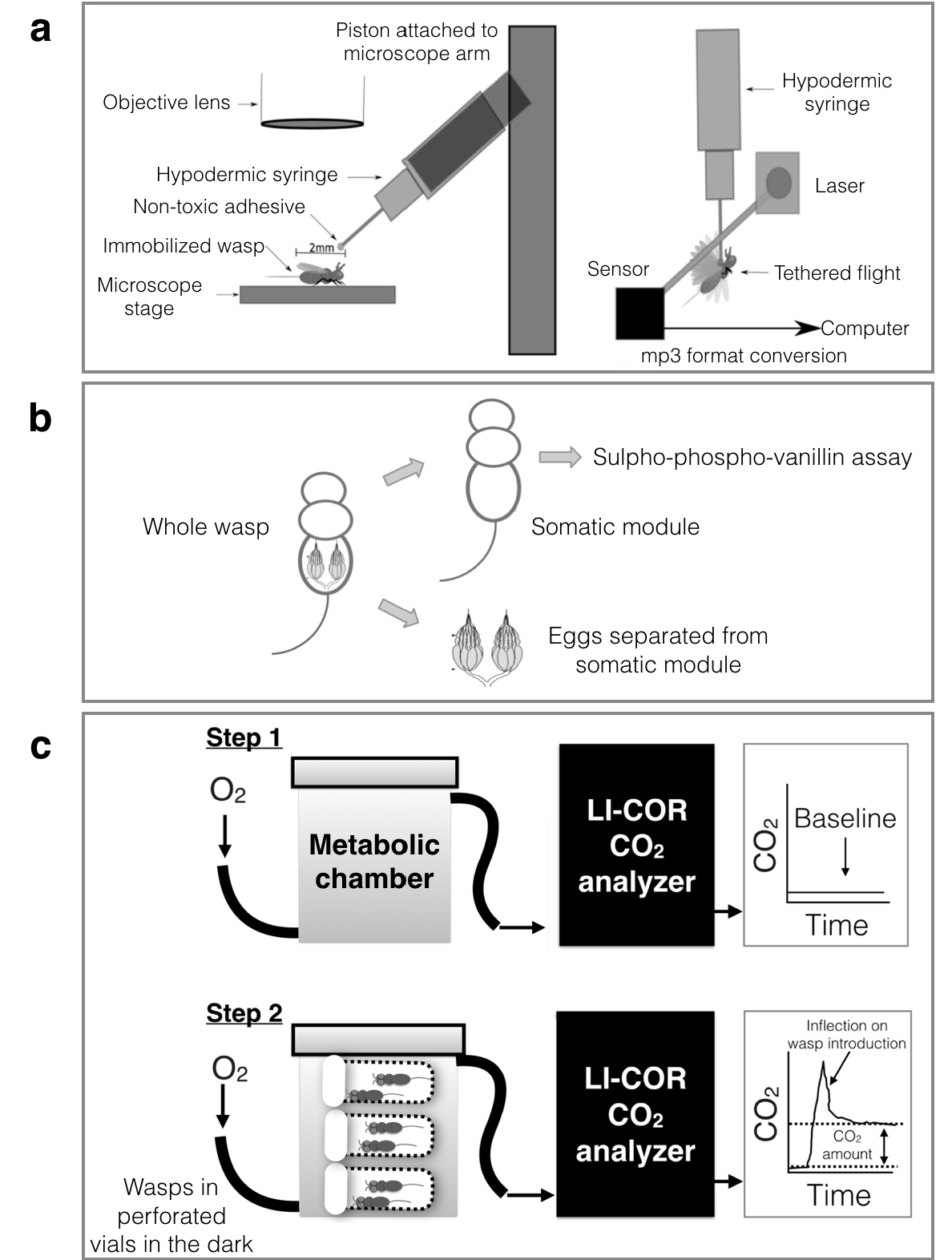


**Figure S4.** Putative phylogenetic relationships of wasp species of *F. racemosa* with branch lengths set to unity. The species abbreviations are the same as in Figure 2 (main text)*.*

**
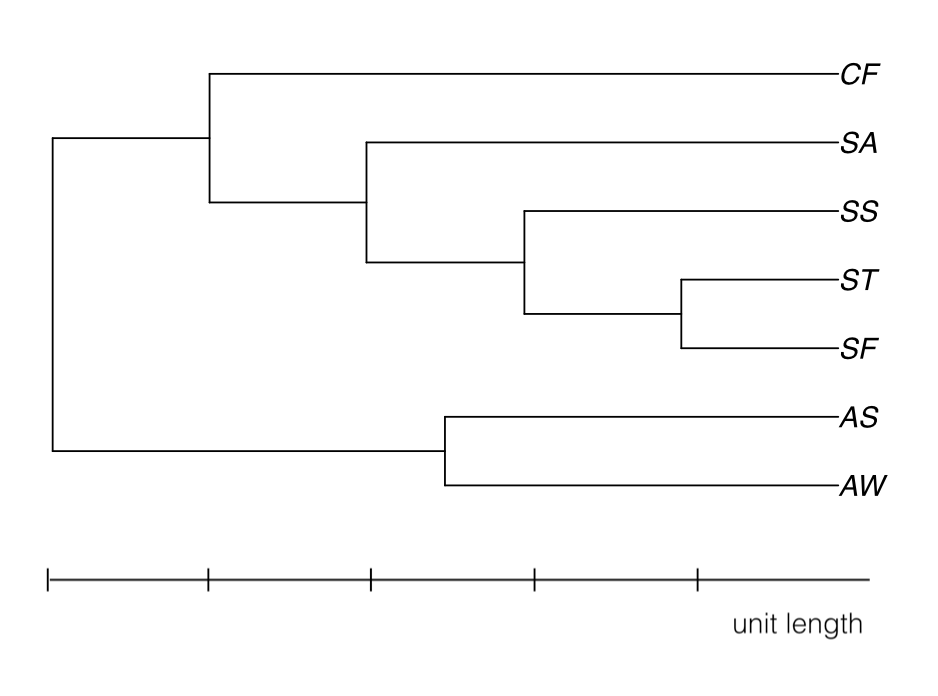
**
